# An evaluation of efficacy of the auto-dissemination technique as a tool for *Aedes aegypti* control in Madeira, Portugal

**DOI:** 10.1186/s13071-019-3454-3

**Published:** 2019-05-03

**Authors:** Gonçalo Seixas, Richard E. L. Paul, Bianca Pires, Gonçalo Alves, Ana de Jesus, Ana-Clara Silva, Gregor J. Devine, Carla A. Sousa

**Affiliations:** 1 UEI Parasitologia Médica, Global Health and Tropical Medicine, GHTM, Instituto de Higiene e Medicina Tropical, IHMT, Universidade Nova de Lisboa, UNL, Rua da Junqueira 100, 1349-008 Lisbon, Portugal; 2 Functional Genetics of Infectious Diseases Unit, Department of Genomes and Genetics, Institut Pasteur, 75015 Paris, France; 3 Centre National de la Recherche Scientifique (CNRS), Génomique évolutive, modélisation et santé UMR 2000, 75724 Paris Cedex 15, France; 4 Unidade de Engenharia Sanitária, Departamento de Promoção e Proteção da Saúde, Instituto de Administração da Saúde e Assuntos Sociais, IP-RAM, Funchal, Madeira Portugal; 5 Mosquito Control Laboratory, QIMR Berghofer Medical Research Institute, Brisbane, QLD Australia

**Keywords:** Auto-dissemination, Pyriproxyfen, Europe, *Aedes*

## Abstract

**Background:**

The frequency and intensity of arboviral epidemics is steadily increasing and posing an intractable public health burden. Current vector control methods are proving ineffectual and despite progress in the development of high technology approaches, there is an urgent need for the development of tools for immediate implementation. Several studies suggest that the auto-dissemination of pyriproxyfen (PPF) is a promising new approach to larviciding although there is little detail on the conditions under which it is optimally effective. Here, we evaluate the efficacy of the approach in urban and rural sites in Madeira, Portugal.

**Results:**

Auto-dissemination of PPF through adapted Biogents Sentinel traps (BGSTs) resulted in a modest but consistent impact on both juvenile and adult mosquito populations, but with considerable spatial heterogeneity. This heterogeneity was related to the distance from the BGST dissemination station as well as the local density of adult mosquitoes. There was evidence that the impact of PPF was cumulative over time both locally and with gradual spatial expansion.

**Conclusions:**

The density of adult mosquitoes and the spatial distribution of dissemination devices are key factors in mediating efficacy. In addition, urban topography may affect the efficiency of auto-dissemination by impeding adult mosquito dispersal. Further studies in a range of urban landscapes are necessary to guide optimal strategies for the implementation of this potentially efficacious and cost-effective approach to larviciding.

## Background

Mosquito-borne infections are a major public health burden. Amongst these, arboviruses are imposing considerable immediate and long-term pathological and socio-economic problems in many countries. The burden of arboviral disease is higher than the combined impact of 17 other conditions, including upper respiratory infections and hepatitis B [1]. Epidemics are complicated by overloaded public health infra-structures, ineffective intervention strategies and collateral economic damage through lost employment and negative impacts on tourism, an industry that many countries rely upon [2].

The management of these diseases in urban settings is particularly difficult as human populations and their associated mosquito habitats have created environments that are almost impossible to treat with traditional insecticidal interventions. *Aedes aegypti*, the major urban vector of arboviruses, is superbly adapted to man-made environments, transmitting dengue, chikungunya and Zika viruses. Uncontrolled, unplanned towns and cities and the detritus of our “throw-away” society form an optimal transmission environment and a major challenge to arbovirus management. The increasing frequency and amplitude of arboviral epidemics even in Europe bears testament to the scale of the threat [3–5].

Despite progress in the development of vaccines for chikungunya, dengue and Zika [6–8], no current candidates are likely to have general application in endemic areas and it is generally agreed that an integrated approach with a significant role for vector control will be needed [9, 10]. Unfortunately, current mosquito control techniques are proving ineffectual and there remains an urgent need for the development of interventions that could be reasonably implemented across the environments and epidemiological contexts that arboviruses inhabit. Achieving sufficient coverage of aquatic habitats, mosquito populations or indoor resting areas by any intervention is proving impossible in many urban transmission settings [11]. Insecticide-based control programmes are also threatened by the evolution and spread of mosquitoes that are resistant to the very limited set of chemistries that we currently rely upon [12].

Pyriproxyfen (PPF) is a WHO-approved pupacide that can be used in drinking water and is recommended for use in conventional larviciding programmes against container breeding mosquitoes such as *Ae. aegypti* [13, 14]. It is a synthetic analogue of juvenile hormone and, at miniscule doses, it prevents larval and pupal development and affects female fertility and male spermiogenesis [15, 16]. The potential of PPF as an auto-dissemination tool has been proven in a variety of small-scale trials in Peru and Italy [11, 17] and more recently at a larger scale in the Amazon [18].

The auto-dissemination of PPF co-opts the innate behaviors of container-breeding mosquitoes to distribute this chemical to their aquatic habitat. Mosquitoes exposed to a surface contaminated with PPF subsequently spread the pupacide to their own breeding sites during oviposition [11, 17]. The exposure of the adult mosquito population is achieved through the use of artificial structures, called dissemination stations, which lure mosquitoes seeking oviposition or resting sites. Contaminated mosquitoes then transport the particles of PPF on their body and legs to the containers that they visit subsequently. This strategy may be particularly effective for *Ae. aegypti* because it may lay its eggs in several sites (skip oviposition), thus allowing greater breeding site coverage [17, 19]. This technique may complement source reduction and larviciding campaigns by efficiently targeting the most productive containers (because the mosquito chooses and then contaminates its own breeding sites). It is potentially a more effective intervention than adult lethal traps, because its impact is amplified between the dissemination devices and the breeding sites - a small number of devices can contaminate a much wider habitat [11, 20].

Here we describe a 2-year study on the efficacy of PPF in reducing populations of *Ae. aegypti* using an auto-dissemination strategy implemented at two contrasting sites, rural and highly urban, in Madeira, Portugal. These studies were conducted in 2015–2016, precipitated by the 2012–2013 dengue outbreak on Madeira that resulted in > 2000 cases [3].

## Methods

This study aimed to assess the efficacy of adapted Biogents-Sentinel traps (Biogents, Regensburg, Germany) for the auto-dissemination of pyriproxyfen in reducing both juvenile and adult *Aedes aegypti* populations in a rural and urban site in Madeira and to assess factors affecting efficacy.

### Evaluation of Biogents Sentinel (BGS) traps as dissemination stations

**Fig. 1:**
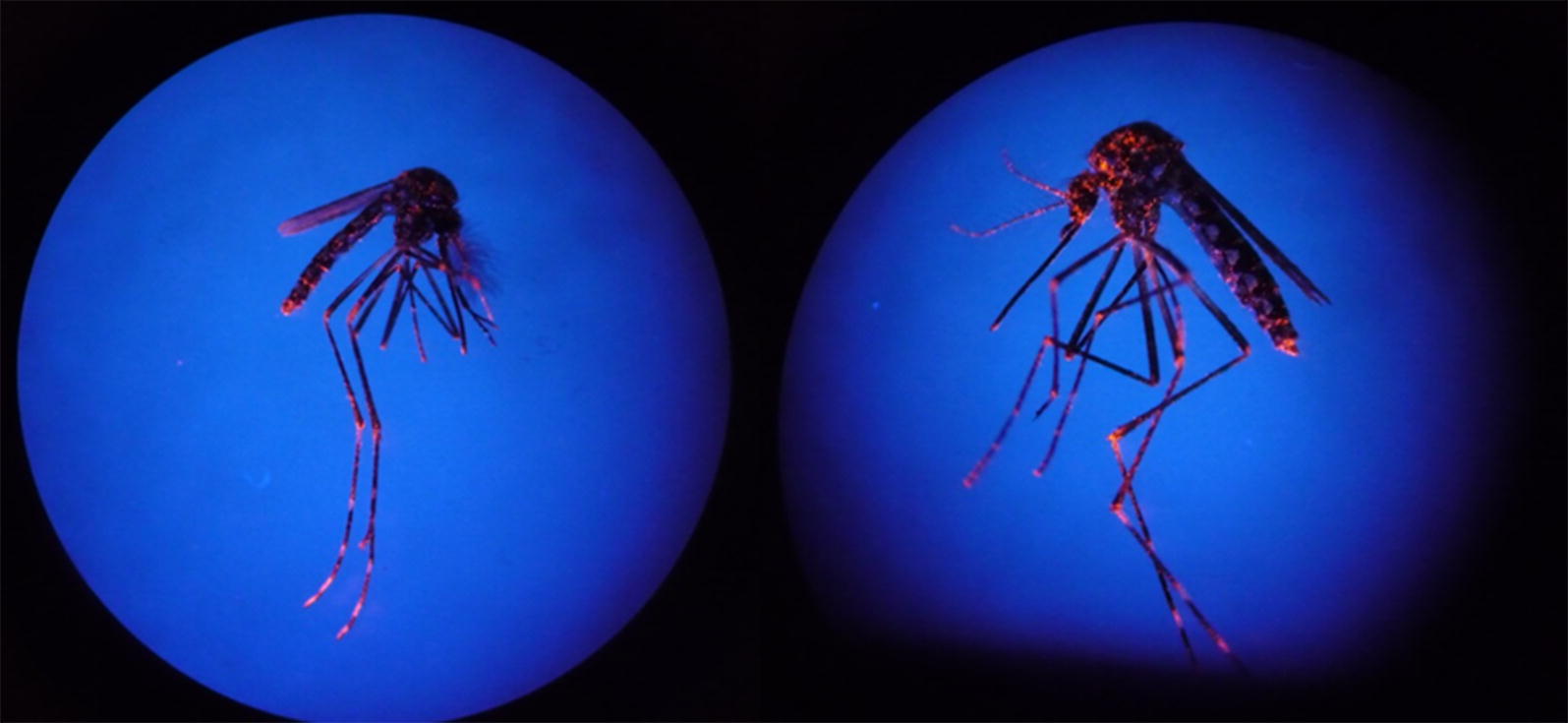
Mosquitoes captured from a room containing an adapted BGST. Their contamination with fluorescent dust demonstrates their exposure and subsequent release from these devices

To ensure the suitability of adapted Biogents-Sentinel traps (BGSTs) for the dissemination of PPF, a small-scale proof of principle was carried-out under laboratory conditions. A prototype had previously been successfully field-tested in Peru (GJ Devine, unpublished data). A BGST with a fine mesh catch bag, but without the cone net that normally impedes escape, working in 1 hour on/off cycles, was placed in an isolated room (9 m^2^, 25 ± 2 °C, 12 h Light:Dark photocycle). The BGST capture bag was first treated with fluorescent dust (DayGlo Color Corp., Cleveland, OH, USA) to simulate PPF particles (20–30 µm in diameter). A total of 50 mosquitoes (25 unfed females and 25 males), Funchal strain, F1 generation, were released into the room. After 24 h, resting mosquitoes were captured individually using mouth aspirators. Contamination with fluorescent dust was observed using a stereomicroscope under a UV light (Fig. 1). All mosquitoes contaminated with dust must have entered, and then escaped from the adapted BGST.

### Auto-dissemination studies

**Fig. 2:**
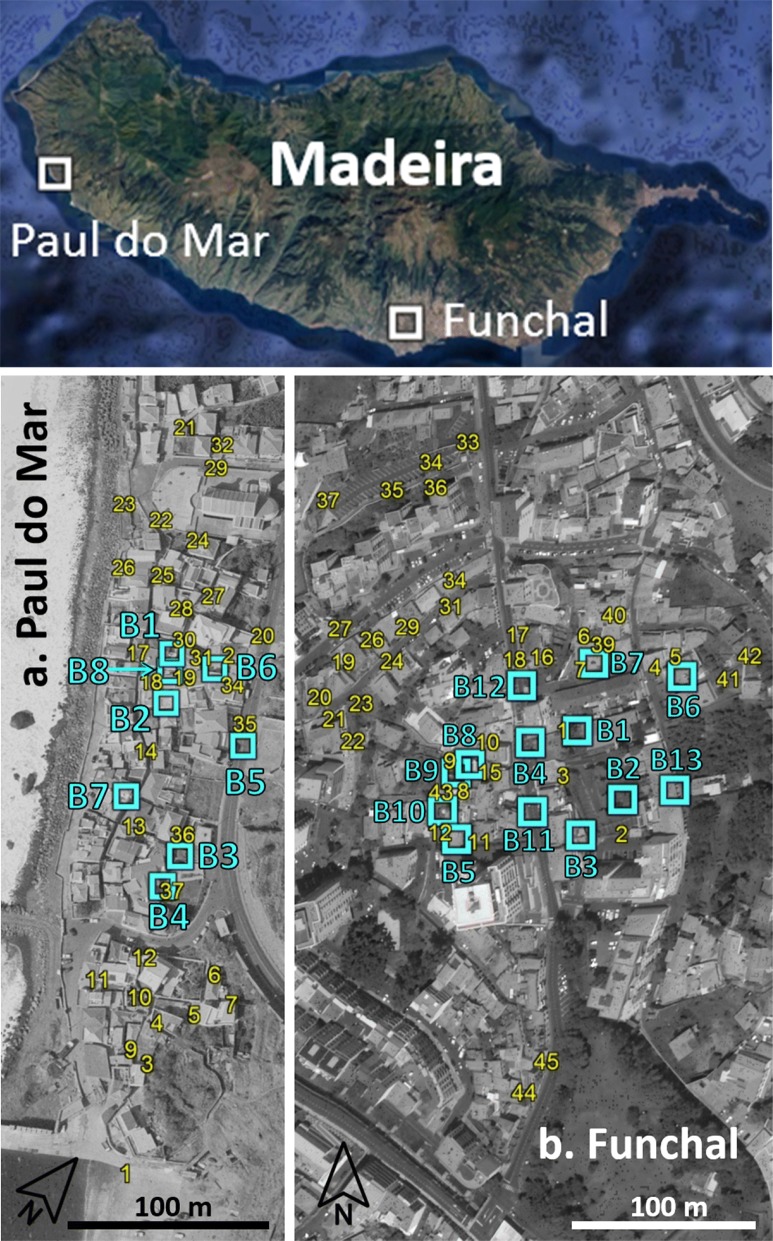
BGST dissemination sites (blue) and ABS (yellow) in Paul do Mar (**a**) and Funchal (**b**)

Two PPF auto-dissemination studies were carried out in Madeira, one in the parish of Paul do Mar, Calheta County (a coastal village with 868 residents), and the other the following year (2015) in the parish of Imaculado Coração de Maria, Funchal (an urban area with 6207 residents). In Paul do Mar, seven adapted BGSTs (powered by battery and described above) were deployed (approximately one per 200 m radius) and 37 artificial breeding sites (ABS), were distributed throughout the study area of 27,138 m^2^ (Fig. 2a). Each ABS consisted of a 12 cm diameter container filled with 250 ml of dechlorinated tap water, a cat food pellet and 20 third-instar larvae of Funchal strain, F1 generation, reared in the insectary. These larvae act as sentinels for the transfer of PPF to the ABS. In Funchal, 13 adapted BGSTs were placed in an area of 125,600 m^2^ (approximately one per 200 m radius) and 45 ABS distributed through a wider area (Fig. 2b). The auto-dissemination study comprised four phases: a pre-treatment phase to measure adult mosquitoes in BGSTs and larval mortality in ABS; two PPF treatment phases (T1 and T2) using the same BGSTs but adapted for auto-dissemination of PPF to measure larval mortality in ABS and a final post-treatment assessment of mosquito density using the BGSTs as in the pre-treatment phase. During the treatment phases, the catch bags of adapted BGSTs were dusted with a 10% PPF formulation. The traps were set to a one hour on/off cycle. The ABS were used to monitor larval mortality in the presence or absence of PPF-treated BGSTs. In each of the treatment phases (pre-treatment, T1, T2 and post-treatment) new ABS were placed in the same locations. Larval development in the ABS was observed at 48 h intervals. All live pupae were transferred to cups of uncontaminated water and taken to the laboratory to record emergence or death. All dead larvae and pupae were also removed and recorded. The ABS were removed when none of the original sentinel cohort remained. We then proceeded to the next phase. The duration of the phases differed according to the rate of immature development and hence the time to the collection of the last pupae in the ABS. In Paul do Mar, the duration of the phases was 6 days (pre-treatment), 9 days (T1 treatment) and 29 days (T2 treatment); in Funchal the durations were 9, 11 and 13 days, respectively. When the treatment phases ended, the BGSTs were fitted with new catch bags (without PPF), the cone funnel was replaced and the traps were run constantly to assess adult mosquito numbers per day for a further week. Independently, ovitraps (14 in Paul do Mar and 78 in Funchal) were monitored throughout the two sites to provide an additional measure of mosquito abundance throughout the year.

There was some loss of ABS in each treatment phase due to interference by domestic animals and on some occasions fewer than 20 larvae were recovered from each ABS suggesting larval death, decomposition and/or cannibalism. Mortality analyses were calculated using only those mature larvae/pupae that were recovered and followed to emergence or death in the laboratory.

### Statistical analyses

In order to compare the effects of PPF auto-dissemination on adult catches pre- and post-treatment using standard BGSTs, we used Generalized Linear Mixed Models (GLMM) using Genstat v. 15 [21] to examine individual BGSTs and overall catches. Period (pre- or post-treatment) was fitted as an explanatory variable and BGSTs as a random variable in a log-linear mixed model. Only totals per period were analysed. The impact of auto-dissemination on juvenile survival in the ABS was analysed by mixed model logistic regression, fitting period (pre-treatment, T1 or T2) as an explanatory factor, individual ABS as a random variable and juvenile mortality as the number of dead juveniles over the total number of collected juveniles per ABS. Only totals per period were analysed for the Paul do Mar study site; temporal information on larval mortality was available for Funchal but only analysed temporally in the SaTScan analysis.

### Spatial cluster analysis

This was performed using Kulldorffʼs scan statistic in SaTScan (v.9.1.1) (http://www.satscan.org/) [22]. A discrete Poisson model was used to analyse the spatial distribution of adult mosquito catches (females and males were combined) in standard, non-adapted BGSTs and larval/pupal mortality in ABS. The program compares the occurrence of adult mosquito catches (or larvae/pupae dead) over the number of sites (BGST or ABS) inside a randomly generated cluster circle compared to the rest of the population. The unit of analysis was the sampling site (BGST or ABS). An infinite number of cluster circles are generated with a maximum diameter set to values between 10–250 m, adapted to the area of study. Clusters represented hot or cold spots representing greater or less than expected adult mosquito numbers or juvenile mortality. Only clusters with no geographical overlap were accepted. A relative risk of observed lower (cold spot) or higher (hot spot) numbers of events (here adult mosquitoes or larval mortality) than expected from the whole study area is calculated and a likelihood ratio test performed. For BGST catches only a spatial analysis was performed. For Funchal, information on larval/pupal mortality was available every 48 h and thus a spatio-temporal analysis was performed. Additional analyses on larval/pupal mortality were performed fitting BGST adult mosquito catches in the nearest BGST as a covariate. A Bonferroni correction was applied when multiple analyses were performed on the same data set.

## Results

### Evaluation of the adapted Biogents Sentinel traps (BGST) as dissemination stations

Of the 50 mosquitoes released in the room, 42 mosquitoes (25 females and 17 males) were recaptured. By exposure to UV light, it was confirmed that all 42 mosquitoes were marked with fluorescent dust (Fig. 1) and had thus visited the BGST, become contaminated with powder and been released.

### Auto-dissemination Paul do Mar

#### BGST adult catches

**Fig. 3:**
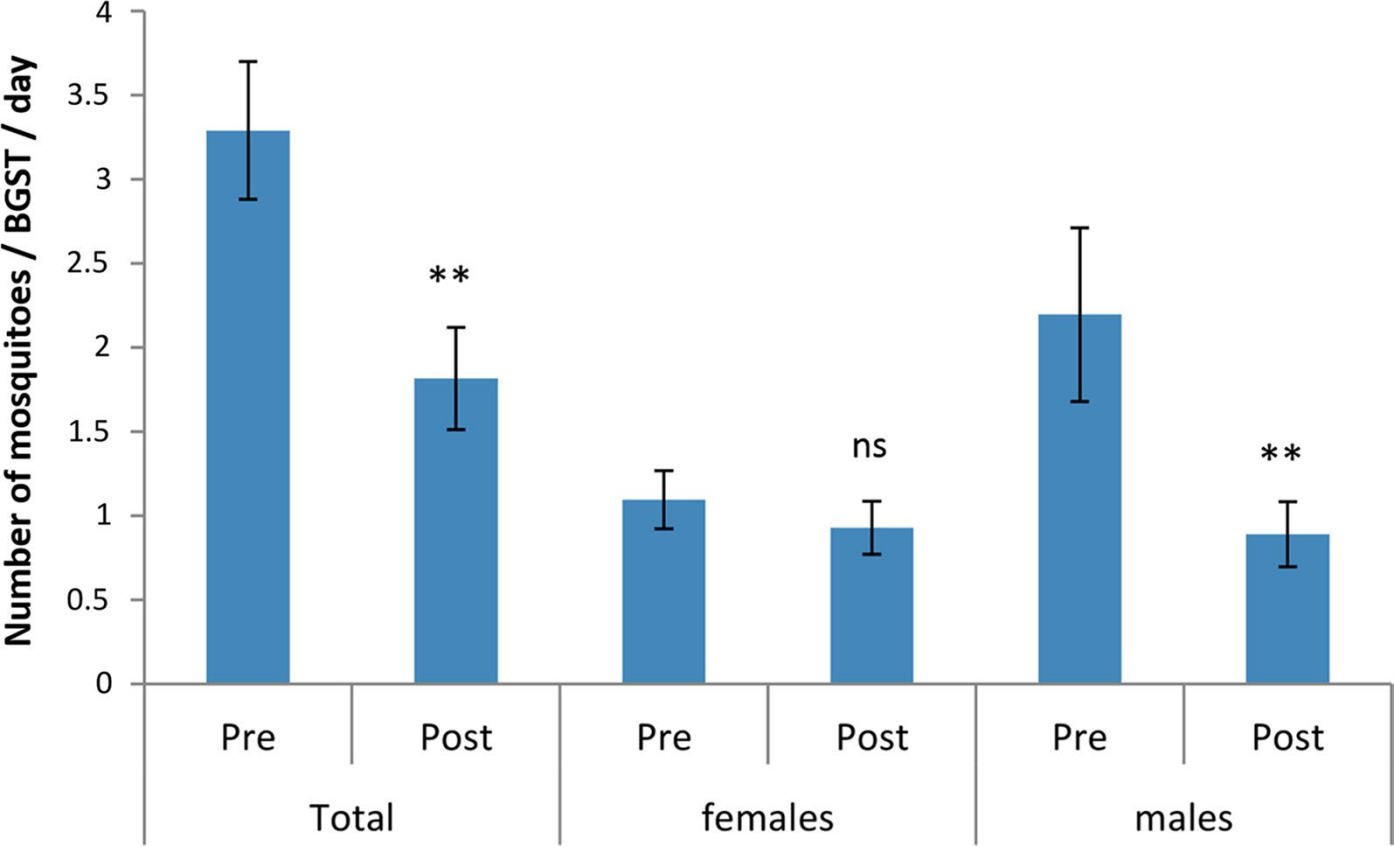
*Aedes aegypti* caught by BGSTs per day in the pre- and post-treatment periods (Paul do Mar site). Mean numbers and standard errors are shown. Significant differences between pre- and post-treatments are noted (ns: not significant, **P < 0.01)

Adult *Ae. aegypti* mosquito numbers captured in the BGSTs (Fig 2a) decreased between the pre-treatment and post-treatment periods, due to a sharp reduction in males (*χ*^2^_1_ = 14.5, *P* = 0.001) (Fig. 3). There was variation among catches from the seven BGSTs, but again only for male mosquitoes (*χ*^2^_6_ = 38.97, *P* = 0.019). The mean number of eggs per surveillance ovitrap per week (over the 14 ovitraps spread in and around the study area) varied in the pre-treatment weeks between 1 and 18 and between 1 and 17 in the post-treatment period, suggesting that overall the female mosquito abundance was low but comparable in the two periods.

Spatial analyses revealed significant heterogeneity in BGST catches, with greater than or less than expected adult numbers in some clusters when compared to the study site overall. This was most notable during the pre-treatment period (Table 1) and largely reflected the high and low mosquito densities observed in specific BGSTs (Fig. 2). These differences were unstable and disappeared during the post-treatment period.

**Table 1:** Spatial hot and cold spots of adult mosquito catches in Paul do Mar

Cluster	BGST	Area radius (km)	Obs.	Exp.	RR	*P*-value
Pre-treatment						
1	4	0	97	25.57	7.1	10^−16^
2	2, 5, 6	0.043	34	76.71	0.31	4.4×10^−11^
3	3	0	10	25.57	0.36	0.002
Post-treatment: no significant clusters						

*Notes*: Shown are the BGST ID numbers associated with the hot and cold spots (with area of hot/cold spots given) and the observed (Obs.) and expected (Exp.) numbers caught from those traps with associated relative risk (RR) and *P*-values

#### Artificial breeding sites (ABS) - impacts on juveniles

**Fig. 4:**
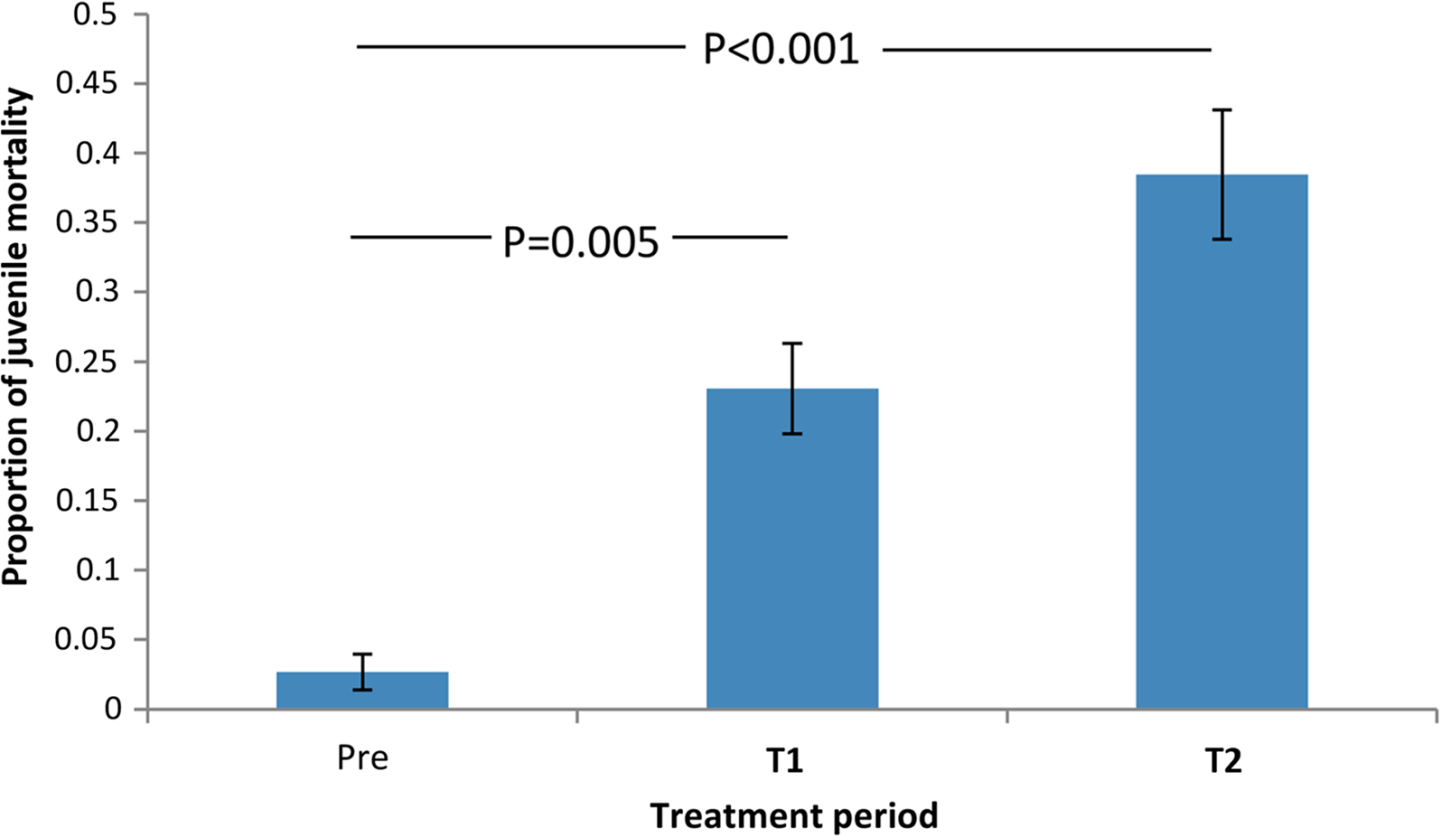
Larval and pupal mortality during pre-treatment, T1 and T2 periods (Paul do Mar site). Proportion refers to the larval and pupal mortality that occurred among the sentinel larval cohorts that were used to seed the artificial breeding sites (ABS). Mean proportions and standard errors calculated from the fitted GLMM model are shown. Significant differences between treatment phases are noted

Thirty-seven ABS each seeded with 20 larvae were distributed throughout the study site (Fig 2a). Mortality rates of juvenile stages (larvae and pupae) were recorded during three periods: pre-treatment, T1 and T2. Juvenile mortality increased from 2.7% (SE 1.3) in the pre-treatment period to 23.1% (SE 3.3) in T1 and 38.4% (SE 4.7) in T2 (*χ*^2^_2_ = 13.07, *P* = 0.002) (Fig. 4). In T1, almost all mortality occurred in ABS located near to BGST dissemination stations. In T2, juvenile mortality was more geographically widespread. There was significant variation in the impact of PPF on ABS: from 0–100% juvenile mortality (Table 2).

**Table 2:** Percent of ABS that had < 5%, 5–50% and > 50% juvenile mortality according to treatment period

	Percentage juvenile mortality		
	0–5%	5–50%	50–100%
Pre-treatment	81.3	18.7	0
T1	54.6	21.2	24.2
T2	26.9	34.6	38.5

To explore whether adult mosquito density affected juvenile mortality in the ABS and contributed to the observed spatial heterogeneity, we analysed the impact of adult mosquito abundance from the nearest adapted BGST to each ABS (using total pre-treatment adult counts for T1 and total post-treatment adult counts for T2). Whilst overall there was no impact of adult mosquito densities on adjacent juvenile mortality rates, there was a near-significant association of higher adult densities with higher T2 juvenile mortality (Log-linear regression *χ*^2^_1_ = 3.65, *P* = 0.068).

Spatial analyses revealed greater and less than expected juvenile mortality in the ABS during pre-treatment and treatment periods (Table 3). A single ABS with 50% mortality was responsible for a hot spot in the pre-treatment period. During the T1 period the spatial variation in mortality rates increased with four distinct hot and cold spots. At the end of the T2 period there remained significant spatial variation but at different sites. Indeed, one cluster that showed zero mortality in the T1 period became a mortality hot spot during the T2 period. There was a notable impact of adult mosquito density on larval and pupal mortality and the significance of the hot and cold spots was reduced or even lost when adult density was fitted as a covariate (Table 3). This suggests that adult mosquito density is contributing to the spatial patterns of mortality in the ABS.

**Table 3:** Hot and cold spots of juvenile mortality in Paul do Mar

Cluster	ABS code	Area radius (km)	Obs.	Exp.	RR	*P*-value
Pre-treatment						
1	14	0	10	0.91	17.7	7.9 × 10^−8^
T1						
1	20–30, 32	0.120	21	58.62	0.24	3.1 × 10^−10^
2	37	0	20	2.82	8.11	5.4 × 10^−10^
3	2, 15, 16, 31, 33–35	0.026	65	28.95	3.32	5.4 × 10^−10^
4	1, 3–5, 10, 11	0.084	0	20.30	0	1.9 × 10^−9^
With BGST pre-treatment adult catches as covariate						
1	1, 3–5, 10, 11	0.084	0	17.00	0	2 × 10^−7^
2	37	0	20	4.00	5.67	5.3 × 10^−7^
T2						
1	3–5, 11, 12, 37	0.058	51	28.50	2.36	0.00063
2	21, 29, 32	0.025	2	15.37	0.12	0.00066
3	15, 34, 36	0.048	7	22.73	0.27	0.0028
With BGST post-treatment catches as covariate: no clusters						

*Notes*: Shown are observed (Obs.) and expected (Exp.) mortality rates (numbers of dead larvae/pupae) in significant hot or cold spots during pre-treatment, T1 and T2 periods, with and without BGST adult mosquito catches fitted as a covariate. ABS ID numbers, area covered by hot/cold spot, and relative risk (RR) with associated *P*-values are presented

### Auto-dissemination Funchal

#### BGST adult catches

**Fig. 5:**
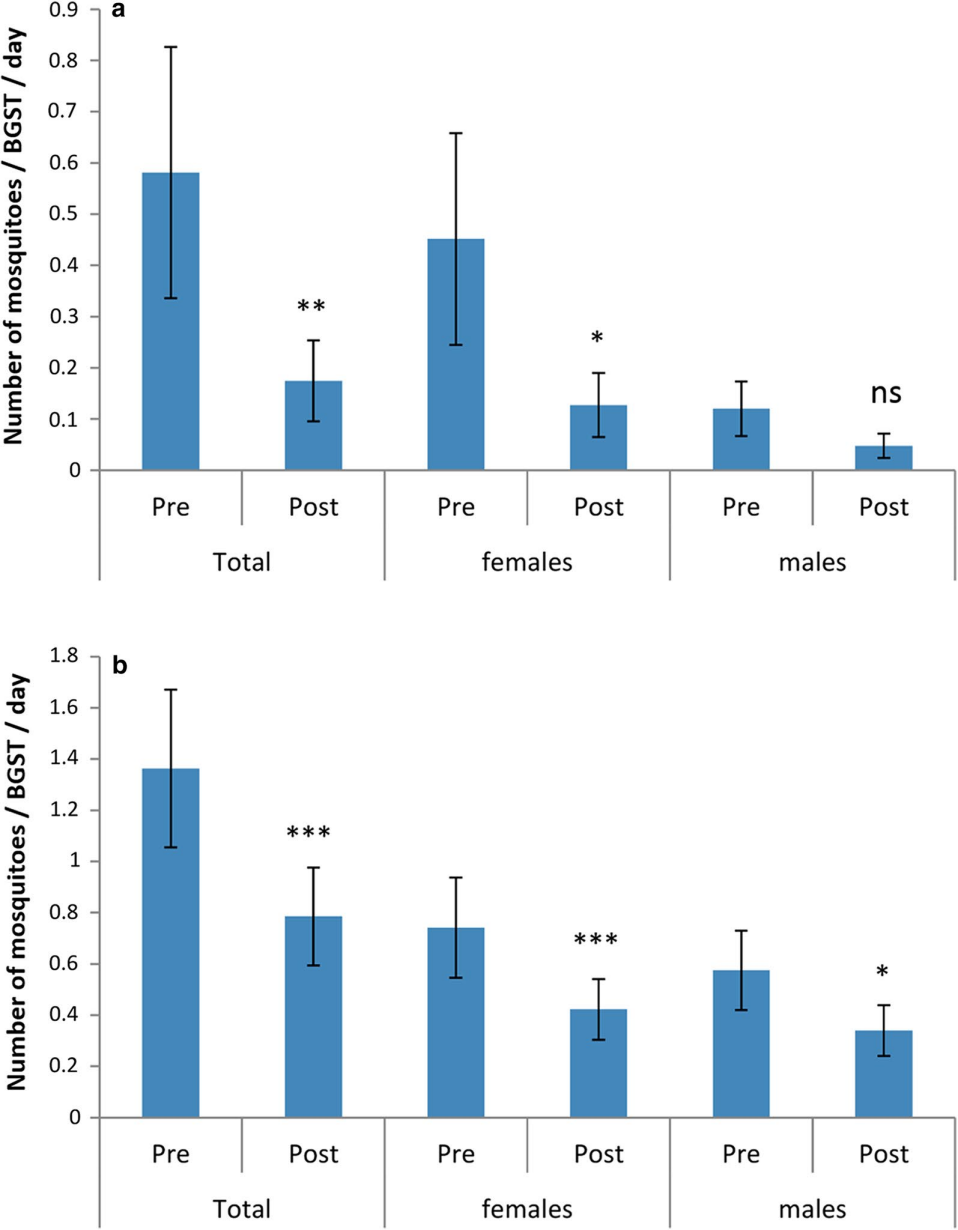
Mosquitoes caught by BGSTs per day in the pre- and post-treatment periods (Funchal site). Mean numbers and standard errors are shown. **a** *Ae. aegypti.* **b** *Culex* spp. Significant differences between pre- and post-treatments are noted (ns: not significant, *P < 0.05, **P < 0.01, ***P < 0.001)

Adult *Ae. aegypti* numbers captured in the BGSTs (Fig 2b) decreased significantly between pre- and post-treatment periods (Total: *χ*^2^_1_ = 9.13, *P* = 0.009; female: *χ*^2^_1_ = 7.46, *P* = 0.015; male: *χ*^2^_1_ = 3.74, *P* = 0.073) (Fig. 5a). In contrast to Paul do Mar where there were almost no *Culex* spp., in Funchal *Culex* spp. mosquitoes were present and also decreased between pre- to post-treatment periods (Total: *χ*^2^_1_ = 23.5, *P* < 0.001; female: *χ*^2^_1_ = 23.1, *P* < 0.001; male: *χ*^2^_1_ = 4.74, *P* = 0.045) (Fig. 5b). There was considerable variation in catch numbers among the thirteen BGSTs (*χ*^2^_12_ = 80.6, *P* < 0.001). The mean number of eggs in the 4 ovitraps sited in the treatment area varied between 60 (pre-treatment) and 50 (post-treatment). In greater Funchal the other 74 ovitraps yielded between 30 eggs per trap (pre-treatment) to 20 (post-treatment). This suggests that overall mosquito abundance remained similar between pre- and post-treatment periods and that observed decreases in adults in the treatment area were not due to a universal temporal effect.

As observed in Paul do Mar, spatial analyses revealed heterogeneity in adult mosquito captures with BGSTs (Table 4). Hot and cold spots were in the same areas for *Aedes* and *Culex*. During the post-treatment phase, the number, importance and size of these clusters was reduced. The inclusion of pre-treatment adult mosquito density as a covariate further reduced spatial heterogeneity in post-treatment catch rates. This suggests that, as found for juvenile mortality rates in Paul do Mar, variation in adult mosquito densities affects the efficiency of PPF dissemination with measurable effects even in the adult mosquito population.

**Table 4:** Spatial hot and cold spots of adult mosquito catches (*Aedes* and *Culex*) pre- and post-treatment in Funchal

Cluster	BGST	Area radius (km)	Obs.	Exp.	RR	*P*-value
*Aedes*						
Pre-treatment						
1	1	0	42	11.60	4.62	1.9 × 10^−11^
2	3–5, 8, 9, 11	0.048	42	69.69	0.45	0.00021
Post-treatment						
1	1, 7, 12	0.043	48	20.10	4.10	8 × 10^−9^
Post-treatment with pre-treatment *Aedes* as covariate						
1	1, 7, 12	0.043	48	31.00	2.23	0.0085
*Culex*						
Pre-treatment						
1	1–3, 13	0.065	72	32.90	4.63	5.2 × 10^−13^
2	4, 5, 8–11	0.062	14	49.40	0.18	1.3 × 10^−11^
Post-treatment						
1	1, 2, 4, 7, 12	0.047	24	12.30	4.80	0.001
Post-treatment with pre-treatment *Culex* as covariate - no clusters detected						

*Notes*: Shown are observed (Obs.) and expected (Exp.) adult mosquito catches with and without BGST pre-treatment catches fitted as a covariate. BGST ID numbers, area covered by the hot/cold spot, relative risk (RR) and associated *P*-values are given

#### Artificial breeding sites (ABS)

**Fig. 6:**
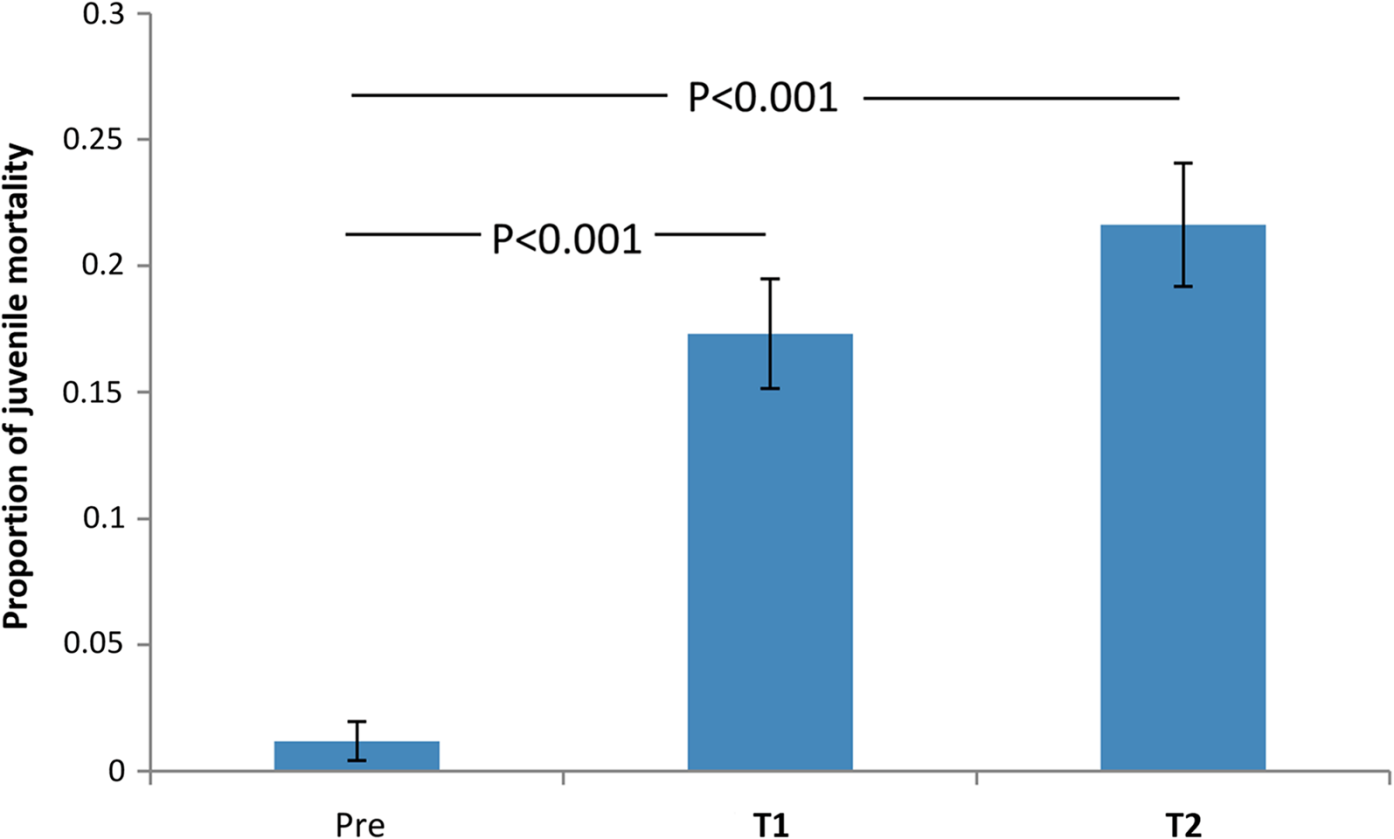
Larval and pupal mortality in ABS in the pre-treatment, T1 and T2 periods (Funchal site). Proportion refers to the larval and pupal mortality that occurred among the sentinel larval cohorts that were used to seed the artificial breeding sites (ABS). Mean proportions and standard errors calculated from the fitted GLMM model are shown. Significant differences between treatments phases are noted

**Fig. 7:**
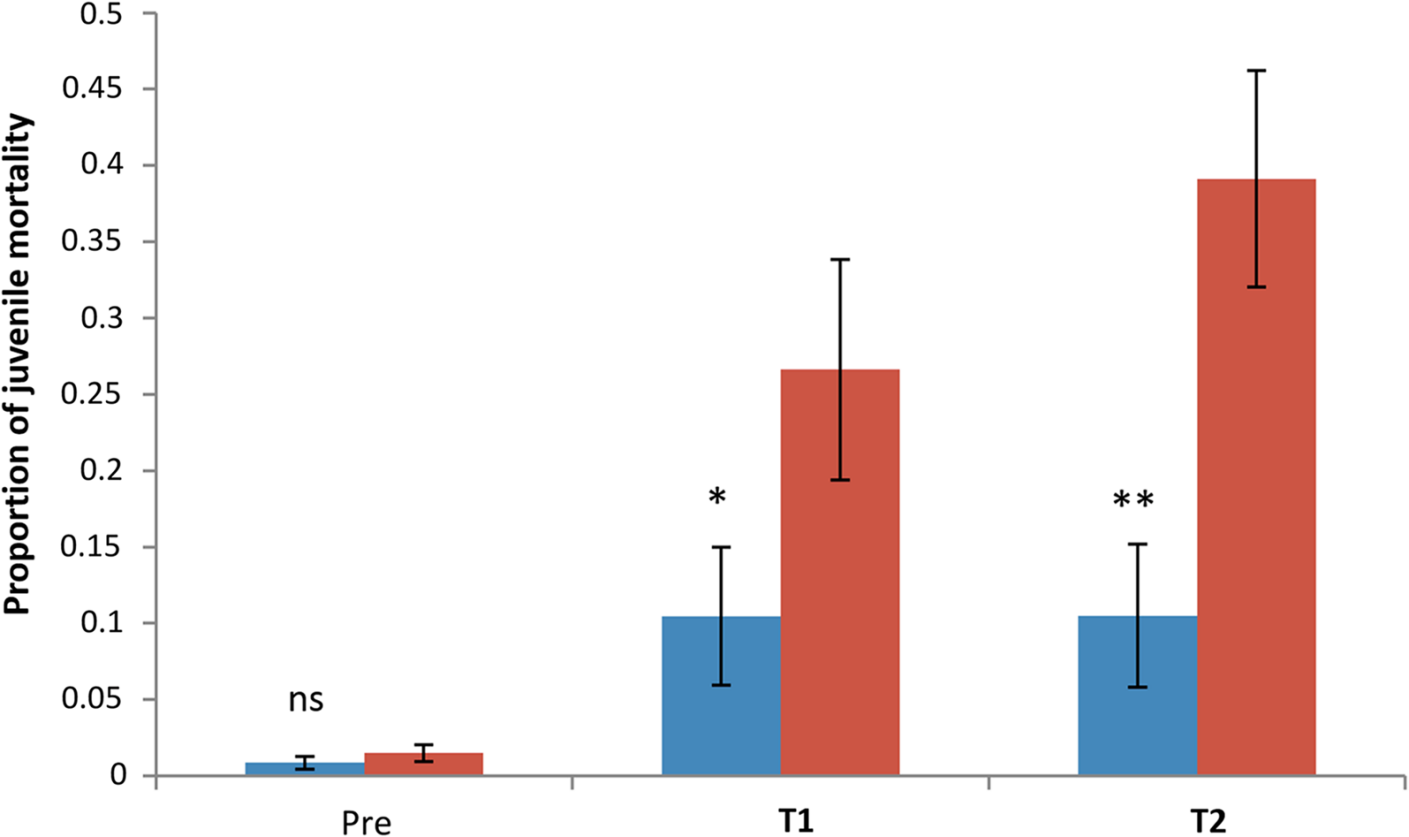
Larval and pupal mortality dependent on distance from dissemination station (Funchal site). Proportion refers to the larval and pupal mortality that occurred among the sentinel larval cohorts that were used to seed the artificial breeding sites (ABS). Mean proportions and standard errors calculated from the fitted GLMM model are shown. Red bars include ABS far from (> 50 m) a dissemination site; blue bars include ABS near to (< 50 m) a dissemination site. Significant differences between near and far sites are noted (ns: not significant, *P < 0.05, **P < 0.01)

Juvenile mortality increased from 1.2% (SE 0.7) in the pre-treatment period to 17.3% (SE 2.2) during T1 and 21.6% (SE 2.4) in T2 (*χ*^2^_2_ = 25.77, *P* < 0.001) (Fig. 6). Despite these significant impacts, many ABS were largely unaffected. Twenty-three of the 43 ABS were < 50 m from a BGST dissemination site (ABS 1–18, 38–43) while the remainder (ABS 19–37, 44–45) were further from dissemination stations (see Fig. 2b). Those ABS that were far from the BGST dissemination sites had significantly lower larval mortality rates in the treatment phases (GLMM Logistic regression of impact of distance, Near *vs* Far, from dissemination site, with individual ABS fitted as a random factor: *χ*^2^_1_ = 11.6, *P* = 0.001; odds ratio 0.22, 95% CI: 0.09–0.52) (Fig. 7).

Spatial analyses of juvenile mortality in the 43 ABS identified no clusters of high or low mortality rates during the pre-treatment phase (Table 5). In T1, by contrast, peak juvenile mortality occurred in an area covering 18 ABS from days 5 to 7 of this treatment phase; these ABS were all close to BGST dissemination stations. There was also one cold spot that included the two ABS (nos 44 and 45) that were far from any dissemination station. The hotspot was again observed during days 1 to 3 of T2 and two new cold spots were identified from day 6 onwards of T2 and involved the ABS furthest from the dissemination stations.

**Table 5:** Juvenile mortality hot and cold spots during the pre-treatment, T1 and T2 periods

Cluster	ABS	Radius (km)	Obs.	Exp.	RR	*P*-value	Day of treatment
Pre-treatment phase							
	Space-time: no clusters						
	Space only: no clusters						
T1 phase							
	Space-time analysis						
1	1, 3, 7–12, 14–18, 22–25, 43	0.088	93	40	6.16	1 × 10^−17^	5 to 7
	Space only analysis						
1	1, 3, 7–12, 14–18, 22–25, 43	0.088	100	61.3	4.06	3.8 × 10^−10^	
2	38, 39	0.0045	0	8.4	0	0.0043	
T2 phase							
	Space-time analysis						
1	1–4, 6–12, 14–18, 38, 39, 43	0.12	55	18.5	3.82	8.5 × 10^−10^	1 to 3
2	27, 33–37	0.086	4	27.2	0.13	1.8 × 10^−5^	6 to 10
3	19–21, 23, 28	0.025	1	13.7	0.07	0.0097	6 to 8
	Space only analysis						
1	1–4, 6–11, 14–18, 38–40, 43	0.11	99	57.8	2.54	6.8 × 10^−8^	
2	34, 36	0.013	4	22.6	0.16	6.6 × 10^−5^	

*Notes*: Shown are observed (Obs.) and expected (Exp.) mortality rates (numbers of dead larvae) in significant hot and cold spots. ABS ID numbers, area covered, relative risk (RR) with associated *P*-values and associated timepoints are given

We assessed the impact of adult abundance (monitored through BGST catches) on the ABS mortality rates. Those ABS distant from BGST dissemination stations (ABS nos 19–37, 44 and 45) were excluded from the analysis, because of the clear negative correlation between distance and juvenile mortality (Fig. 7). Incorporation of adult catches as a covariate of ABS mortality explained the majority of the spatial variation in juvenile survival at local scales. There remained only one “unexplained” coldspot during the early stages of treatment phase 1 (Table 6). This lends further support to the importance of adult mosquito density in efficiently disseminating PPF.

**Table 6:** Juvenile mortality hot and cold spots in ABS proximal to BGSTs

Cluster	ABS	Radius (km)	Obs.	Exp.	RR	*P*-value	Day of treatment
T1 phase							
	Space only, no covariate						
1	38, 39	0.0045	0	7.5	0	0.0043	
	Space with BGST pre count as covariate: no cluster						
	Space-time no covariate						
1	6, 16–18	0.021	24	5.9	5.23	2.8 × 10^−6^	5
2	1, 8–12, 14, 15, 43	0.051	0	9.8	0	0.0035	6
	Space-time with BGST pre count as covariate						
1	9, 10, 18	0.082	0	12.1	0	4.7 × 10^−4^	3 to 4
T2 phase							
	Space only, no covariate: no clusters						
	Space with BGST post count as covariate: no cluster						
	Space-time no covariate						
1	1, 4–7, 16, 38–42	0.065	84	28.2	3.98	1 × 10^−17^	7
2	8–10, 43	0.120	0	30.5	0	3.6 × 10^−12^	7
	Space-time with BGST post count as covariate: no cluster						

*Notes*: Shown are observed (Obs.) and expected (Exp.) mortality rates (numbers of dead larvae/pupae) in hot and cold spots with and without adult densities from closest BGST catch points fitted as a covariate. ABS ID numbers, area covered and relative risk (RR) with associated *P*-values are given. For space-time clusters, associated treatment day is also shown

## Discussion

Despite continued interest in the auto-dissemination concept as a complementary, potentially highly efficient larviciding tool, we have a very limited understanding of how to optimize its operation and deployment. One of the largest scale trials yet conducted [18] set 1000 simple dissemination stations at an approximate density of 1 every 100 meters (1/10,000 m^2^). Distance between these stations and sentinel larval habitats was not recorded although at least some sentinel habitats were clearly placed in the immediate vicinity of the dissemination stations. No direct measure of impact on adult mosquito density was made. We explicitly evaluated the efficacy of adapted BGSTs as auto-dissemination stations for PPF when placed at a low density (approximately 1 every 200 meters). The relationship between their impact and their proximity to sentinel habitats was carefully recorded for a short period of time, in areas of low adult *Ae. aegypti* abundance on the island of Madeira, Portugal.

As shown by others [11, 17, 18], brief deployment of PPF reduced juvenile mosquito survival and impacts appeared to accumulate during continuous deployment. We also measured the impact on the adult mosquito population and found a significant reduction of males and females. Larvicide/pupacide efficacy was higher during the trial conducted at higher mosquito abundance (Paul do Mar), but the impact on the adult mosquito population was lower than in the low abundance setting of Funchal, where there was also a measurable impact on *Culex* spp. adults. It is conceivable that the abundant *Culex* spp. population was compensating for the low density of the target species, *Ae. aegypti*, in Funchal, leading to dissemination of PPF to natural oviposition sites other than our ABS, which were not designed to measure impacts on *Culex* spp. Across all trials there was distinct spatial heterogeneity in the impact of PPF dissemination on juvenile mortality rates and adult abundance. Spatial variation in juvenile mortality decreased over time, suggesting that impact became more universal as the number of dissemination events and ABS coverage increased. Notably, spatial variation in juvenile mortality became negligible once spatial variation in adult abundance was taken into account (i.e. dissemination impacts are related to adult density and, presumably, the number of contamination events). The higher juvenile mortality rates and higher adult densities in Paul do Mar also suggest that dissemination works better when there are more adult mosquitoes. Proximity to a dissemination site also had a very significant effect on juvenile mortality over the short and long terms, suggesting a significant influence of urban topography and mosquito dispersal on dissemination and coverage.

The main challenge in the implementation of vector control measures is to achieve sufficient coverage of the mosquito population (i.e. aquatic habitats treated, houses sprayed, LLINs used). Although source reduction and the application of larvicides is a key tenet of urban mosquito control [23], it is challenging in highly urbanized areas because of the difficulty in identifying and treating myriad aquatic habitats. The auto-dissemination technique, in which mosquitoes contaminate their own aquatic habitats through their resting and oviposition behaviours is a potentially powerful way of overcoming those challenges [11].

Our trials continue to demonstrate this potential but highlight, for the first time, the barriers to successful optimization. Local structural topography will impact significantly on adult mosquito flight range and hence PPF dispersal capacity. The limited flight range of *Ae. aegypti* is well described and further constrained by the urban landscape [24]. This urban heterogeneity will have a significant impact upon the efficacy of PPF delivery to the aquatic habitat and needs to be considered when optimizing the design of auto-dissemination trials.

Target species abundance, the existence of non-targets co-opted into the auto-dissemination process, distance between aquatic sites and dissemination stations, and urban topography will all contribute to the substantial variation in efficacy noted between our results and those reported in other urban trials [18]. A limitation of all auto-dissemination studies to date is the use of sentinel juvenile habitats to monitor efficacy. When measuring impacts in these habitats alone, we have no idea whether the observed impacts underestimate the true power of the technique (are most contaminated adults choosing sites other than those being monitored?). Our study is the first to have demonstrated an impact on adult abundance, the ultimate and most important entomological measure of impact.

The extent to which the auto-dissemination technique is limited by the efficacy of dissemination tools, their spatial deployment patterns in urban environments, and their impact on the productivity of aquatic habitats and adult populations, needs to be addressed before the utility and cost-efficacy of the paradigm can be fully assessed. In particular, we need some universal algorithm for deployment across endlessly variable urban habitats. Numerous studies have addressed the effect of urbanization on mosquito dispersal and population dynamics [25] but, in brief, many facets of the mosquito life-cycle (mating, resting, oviposition, biting rate, survival) are directly influenced by the urban environment (topography, infra-structure, housing). In short, there can be considerable very localized heterogeneity in the suitability of the urban environment for mosquito production, mosquito dispersal, and therefore auto-dissemination as a control measure.

In addition to the problems posed by urban topography, consideration of the attractiveness and transfer efficiency of the dissemination stations is needed [26]. There are currently many experimental and commercial devices available, but these need to be evaluated under comparable field conditions. Our choice of adapted BGSTs as a dissemination device, and of a uniform 10% PPF formulation (specifically manufactured for the purpose of auto-dissemination trials) was an attempt to remove the idiosyncrasies of hand-milling 0.5 WG formulations and improve on the use of roughly treated buckets as dissemination stations [11, 18]. The BGST is widely perceived as the most effective trapping tool on the market, which suggests that our adapted version should be effective as a “lure and release” device.

Furthermore, because it is the adult mosquito that transmits the pathogens, measures of impact on adult mosquito populations are necessary. Our trial demonstrated modest but consistent decreases in adult abundance at both trial sites. This is a crucial first proof prior to implementation of more extensive epidemiological studies that will ascertain the entomological and epidemiological efficacy of auto-dissemination.

## Conclusions

Whilst the complexity of systems seems overwhelming, there is consistent evidence that auto-dissemination of PPF works. The next steps should focus on improving implementation, with its use at the right place and the right time. There has been a call for more intelligent use of currently effective insecticides, and auto-dissemination is a clear example. Optimal intervention strategies will vary from site to site and the combination of auto-dissemination with other interventions is likely. To date, almost all auto-dissemination studies focus on PPF because of its mammalian safety and its unique toxicity at parts per trillion but, in the future, other agents such as insect specific viruses that can infect aquatic life stages [27] or other biological control agents [28] may be considered.

## Abbreviations

BGST: Biogents Sentinel traps
PPF: pyriproxyfen
ABS: artificial breeding sites
GLMM: Generalized Linear Mixed Models
CI: confidence interval
